# Integrated biomarkers and cardiac phenotypes associated with atrial fibrillation: evidence from real-world hospital data

**DOI:** 10.3389/fcvm.2026.1821154

**Published:** 2026-05-15

**Authors:** Xuli Chen, Yanxi Ning, Yuxiang Wang, Yuelin Hu, Yanchao Liu, Wenwen Xiao

**Affiliations:** 1 Department of Electrocardiology, The Second Affiliated Hospital of Wannan Medical University, Wuhu, China; 2 School of Pharmacy, Wannan Medical University, Wuhu, China; 3 Department of Medical Service, The Second Affiliated Hospital of Wannan Medical University, Wuhu, China; 4 Eastern Theater Command Centers for Disease Control and Prevention, Nanjing, China

**Keywords:** atrial fibrillation, AUC, B-type natriuretic peptide, left ventricular ejection fraction, receiver operating characteristic

## Abstract

**Background:**

This study aimed to investigate the association between the B-type natriuretic peptide (BNP)/ left ventricular ejection fraction (LVEF) and atrial fibrillation (AF) and to evaluate its discriminative performance in a real-world hospitalized population.

**Methods:**

We conducted a hospital-based observational study that included 270 consecutive patients hospitalized at the Second Affiliated Hospital of Wannan Medical University between August 2023 and December 2025. BNP/LVEF was calculated by dividing plasma BNP levels by LVEF measured by transthoracic echocardiography. AF was diagnosed based on cardiac rhythm recordings during hospitalization or physician diagnosis. We used a multivariate logistic regression model to analyze the association between BNP/LVEF and atrial fibrillation. Restricted cubic spline analysis was used to explore potential non-linear relationships. Receiver operating characteristic (ROC) curves were used to assess discriminatory performance, and pre-specified subgroup analyses were performed to evaluate effect modification.

**Results:**

Among 270 patients, 123 (45.6%) had atrial fibrillation. In all adjusted models, higher BNP/LVEF values were independently associated with an increased risk of atrial fibrillation in fully adjusted model (OR, 1.02; 95% CI, 1.01–1.04). Restricted cubic spline analysis revealed a significant non-linear association (P-nonlinear 0.001), with a sharp increase in the risk of atrial fibrillation at lower BNP/LVEF levels, followed by a plateau. BNP/LVEF demonstrated good discriminatory ability for atrial fibrillation (AUC = 0.84) in the full unadjusted model. Subgroup analysis showed significant heterogeneity across sex, smoking, alcohol consumption, and coronary heart disease status.

**Conclusion:**

BNP/LVEF is independently and nonlinearly associated with atrial fibrillation and demonstrates good discriminative performance. This integrative marker may provide clinically useful information for AF risk stratification in hospitalized patients.

## 1 Introduction

Atrial fibrillation (AF) is the most common sustained cardiac arrhythmia encountered in clinical practice and represents a growing global public health challenge (1). AF affects tens of millions of individuals worldwide, with recent estimates indicating roughly 60 million prevalent cases and a substantial number of incident cases annually (2). Its absolute burden has increased markedly over the past decades, driven largely by population aging and the rising prevalence of cardiovascular risk factors (3). This arrhythmia is associated with significant morbidity and mortality, including an elevated risk of stroke, heart failure, cognitive decline, and all-cause mortality, contributing to considerable healthcare utilization and economic burden (4). Given the often-asymptomatic nature of AF and its serious clinical sequelae, early identification and effective monitoring strategies, including the investigation of clinical biomarkers and echocardiographic phenotypes, are essential for risk stratification and improved management (5).

B-type natriuretic peptide (BNP), a cardiac neurohormone released in response to ventricular wall stress, is a well-established biomarker for cardiac dysfunction and cardiovascular risk stratification (6, 7). Elevated BNP levels have been associated with adverse cardiovascular outcomes independent of left ventricular ejection fraction (LVEF) in various clinical settings (8, 9). When interpreted alongside echocardiographic indices, they contribute to refined phenotyping of cardiac function and remodeling (10). BNP concentrations are influenced by both atrial and ventricular pathology and have been linked with left atrial remodeling and atrial fibrillation in observational cohorts (11). LVEF, a widely used measure of systolic function, reflects the contractile capacity of the left ventricle and is a cornerstone of echocardiographic assessment (12). While each marker provides important but distinct information, their integration may capture complementary aspects of cardiac structure and function (13). Combined measurement of natriuretic peptides and LVEF has shown improved risk stratification beyond either parameter alone in cardiovascular disease (14). These considerations suggest that a composite index incorporating BNP and LVEF may offer enhanced insight into the burden and mechanistic underpinnings of atrial fibrillation that are not evident when either is evaluated in isolation.

Despite extensive research on BNP and LVEF individually in cardiovascular disease, their combined utility in characterizing atrial fibrillation remains poorly defined (15). Prior studies have demonstrated that elevated B-type natriuretic peptide levels are associated with atrial remodeling and adverse outcomes in AF, and that LVEF provides complementary information on cardiac systolic function (16, 17). However, most investigations have considered these indices in isolation rather than as an integrated measure of cardiac stress and function. BNP levels have been shown to relate to AF progression and outcomes, yet evidence on how the interplay between circulating natriuretic peptides and left ventricular performance influences AF risk is limited, particularly in real-world clinical populations. Therefore, based on real-world hospital data, we examined the association between the BNP/LVEF ratio and atrial fibrillation, aiming to clarify its potential value for risk stratification and mechanistic insight.

## 2 Method

### 2.1 Study population

The study population consisted of hospitalized patients identified from hospital records. For the present analysis, only patients with complete data on BNP, echocardiographic parameters (including LVEF), atrial fibrillation status, and all covariates of interest were included. Patients with missing data for any of these variables were excluded during dataset construction. Therefore, all statistical analyses were conducted using a complete-case dataset.

This study comprised a real-world data of 270 consecutive patients hospitalized at the Second Affiliated Hospital of Wannan Medical College between August 2023 and December 2025. Eligible patients admitted during the study period were enrolled at the time of hospitalization. All participants provided written informed consent prior to inclusion. The study protocol was approved by the Institutional Ethics Committee of the Second Affiliated Hospital of Wannan Medical College (Approval No. WYEFYLS2026007) and was conducted in accordance with the Declaration of Helsinki.

### 2.2 Exposures

The exposure of interest was the ratio of B-type natriuretic peptide to left ventricular ejection fraction (BNP/LVEF). Plasma B-type natriuretic peptide was measured using standardized laboratory assays as part of routine clinical care. Left ventricular ejection fraction (LVEF) was assessed by transthoracic echocardiography as part of routine clinical evaluation. Standard apical four-chamber and two-chamber views were acquired, and LVEF was quantified using the biplane method of disks (modified Simpson's rule) according to guideline-based chamber quantification recommendations. End-diastole was defined as the frame with the largest left ventricular cavity volume, and end-systole was defined as the frame with the smallest left ventricular cavity volume. LVEF was calculated as (end-diastolic volume—end-systolic volume)/end-diastolic volume×100% and recorded as a percentage. The BNP/LVEF ratio was calculated by dividing BNP levels by LVEF to integrate circulating biomarker information with cardiac functional phenotypes.

### 2.3 Outcomes

The primary outcome was atrial fibrillation (AF). AF was defined as documentation of atrial fibrillation on rhythm recordings, including 12-lead electrocardiography, single-lead electrocardiography, continuous in-hospital telemetry, 24–48 h Holter monitoring, or device interrogation, with the tracing reviewed by a physician. AF was also considered present when it was recorded by the treating physician in the electronic medical record or discharge summary, provided that the diagnosis was supported by available rhythm documentation in the hospital chart. In line with guideline-based practice, an AF episode lasting ≥30 s on rhythm monitoring was considered diagnostic when such documentation was available. Outcome ascertainment was performed using hospital records and routine clinical investigations obtained during the index hospitalization.

### 2.4 Covariates

Covariates included age, sex, smoking, alcohol use, diabetes, and coronary heart disease (CHD). These variables were obtained at baseline from the outpatient interview and the standard admission questionnaire and were cross-checked against the electronic medical record when available. Diabetes and CHD were recorded as present if the patient reported a prior diagnosis or if a physician diagnosis was documented in the medical record. For analysis, age was treated as a continuous variable and the remaining covariates as categorical indicators.

### 2.5 Statistical analysis

Continuous variables are presented as mean ± standard deviation and categorical variables as n (%). Between-group comparisons were performed using the Student's *t*-test for continuous variables and the *χ*^2^ test for categorical variables. The primary exposure, BNP/LVEF, was examined both as a continuous variable and as quartiles (Q1–Q4; Q1 as reference). Associations between BNP/LVEF and AF were estimated using multivariable logistic regression models reporting odds ratios (ORs) with 95% confidence intervals (CIs). Four prespecified models were fitted: Model 1, unadjusted; Model 2, adjusted for age and sex; Model 3, additionally adjusted for smoking and alcohol use; and Model 4, further adjusted for diabetes and coronary heart disease. A restricted cubic spline (RCS) analysis with 3 knots was used to assess the dose-response relationship and to evaluate possible nonlinearity. Nonlinearity was formally tested by comparing the spline model with a linear model using a likelihood-ratio test. Discriminative performance was assessed with receiver operating characteristic (ROC) curves and area under the curve (AUC) estimates. Predefined subgroup analyses were performed by age, sex, smoking status, alcohol use, diabetes, and CHD. For each subgroup we fitted stratified logistic models and assessed effect modification by including the product term between BNP/LVEF and the subgroup variable in the full multivariable model. Interaction was summarized by the P-interaction from the product term. A *P*-value less than 0.05 was considered statistically significant. All analyses were based on R 4.5.2.

## 3 Results

### 3.1 Basic characteristics of patients

A total of 270 hospitalized patients were included, of whom 123 (45.6%) had atrial fibrillation (Table 1). Compared with patients without AF, those with AF were older (75.20 vs. 63.70 years), had a substantially higher prevalence of CHD (61.8% vs. 15.6%), markedly higher BNP levels (1796.23 vs. 423.13 pg/mL) and lower LVEF (57.06% vs. 64.56%). The distributions of sex, smoking, alcohol drinking and diabetes did not differ significantly between groups.

**Table 1:** Basic information about hospitalized patients.

Characteristics	Overall	Non- AF	AF	*P*-value
*N*	270	147	123	
Age [mean (SD)]	68.94 (12.46)	63.70 (11.46)	75.20 (10.60)	<0.001
Sex (%)				
Male	125 (46.3)	62 (42.2)	63 (51.2)	0.173
Female	145 (53.7)	85 (57.8)	60 (48.8)	
Smoking (%)				0.223
Yes	25 (9.3)	17 (11.6)	8 (6.5)	
No	245 (90.7)	130 (88.4)	115 (93.5)	
Drinking (%)				0.14
Yes	24 (8.9)	17 (11.6)	7 (5.7)	
No	246 (91.1)	130 (88.4)	116 (94.3)	
Diabetes (%)				0.256
Yes	42 (15.6)	19 (12.9)	23 (18.7)	
No	228 (84.4)	128 (87.1)	100 (81.3)	
CHD (%)				<0.001
Yes	99 (36.7)	23 (15.6)	76 (61.8)	
No	171 (63.3)	124 (84.4)	47 (38.2)	
BNP [mean (SD)]	1,048.66 (2,427.94)	423.13 (1,250.35)	1,796.23 (3,176.78)	<0.001
LVEF [mean (SD)]	61.14 (7.76)	64.56 (4.84)	57.06 (8.59)	<0.001

AD, standard deviation; CHD, coronary heart disease; BNP, N-terminal pro-B-type natriuretic peptide; LVEF, left ventricular ejection fraction.

### 3.2 Associations between BNP/LVEF and atrial fibrillation

We used multivariable logistic regression to study the relationship between BNP/LVEF values and AF (Table 2). In the unadjusted model (Model 1), each unit increase in BNP/LVEF was associated with a 4% increase in the odds of AF (OR, 1.04, 95% CI, 1.02–1.06). This association remained significant after adjusting for age and sex (Model 2: OR, 1.02, 95% CI, 1.01–1.04), and persisted following further adjustment for smoking and alcohol consumption (Model 3: OR, 1.02, 95% CI, 1.01–1.04). The association remained robust after additional adjustment for diabetes and CHD in the fully adjusted model. (Model 4: OR, 1.02, 95% CI, 1.01–1.04).

**Table 2:** Associations between BNP/LVEF and atrial fibrillation.

Models	OR (95% CI)	*P*-value
Model 1	1.04 (1.02–1.06)	<0.001
Model 2	1.02 (1.01–1.04)	0.005
Model 3	1.02 (1.01–1.04)	0.003
Model 4	1.02 (1.01–1.04)	0.027

OR, odd ratio; CI, confidence interval. Model 1: not adjusted. Model 2: adjusted for age and sex. Model 3: further adjusted for smoking and drinking. Model 4: further adjusted for diabetes and CHD.

**Figure 1:**
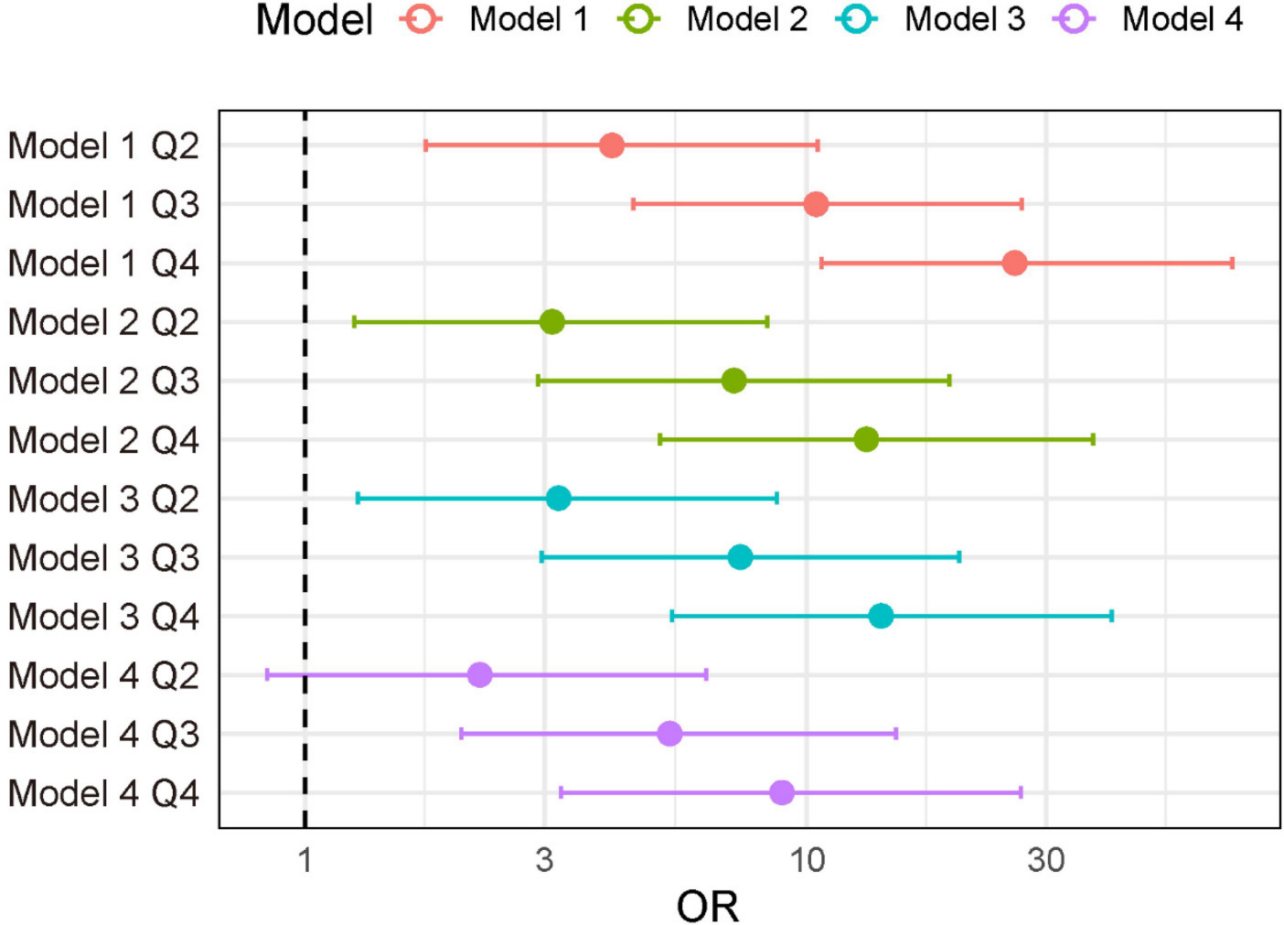
Forest plot of the associations between BNP/LVEF quartiles and atrial fibrillation.

As illustrated in the forest plot (Figure 1), higher BNP/LVEF values were consistently associated with increased odds of prevalent AF across all models, indicating a stable and independent relationship.

### 3.3 Nonlinear relationship

**Figure 2:**
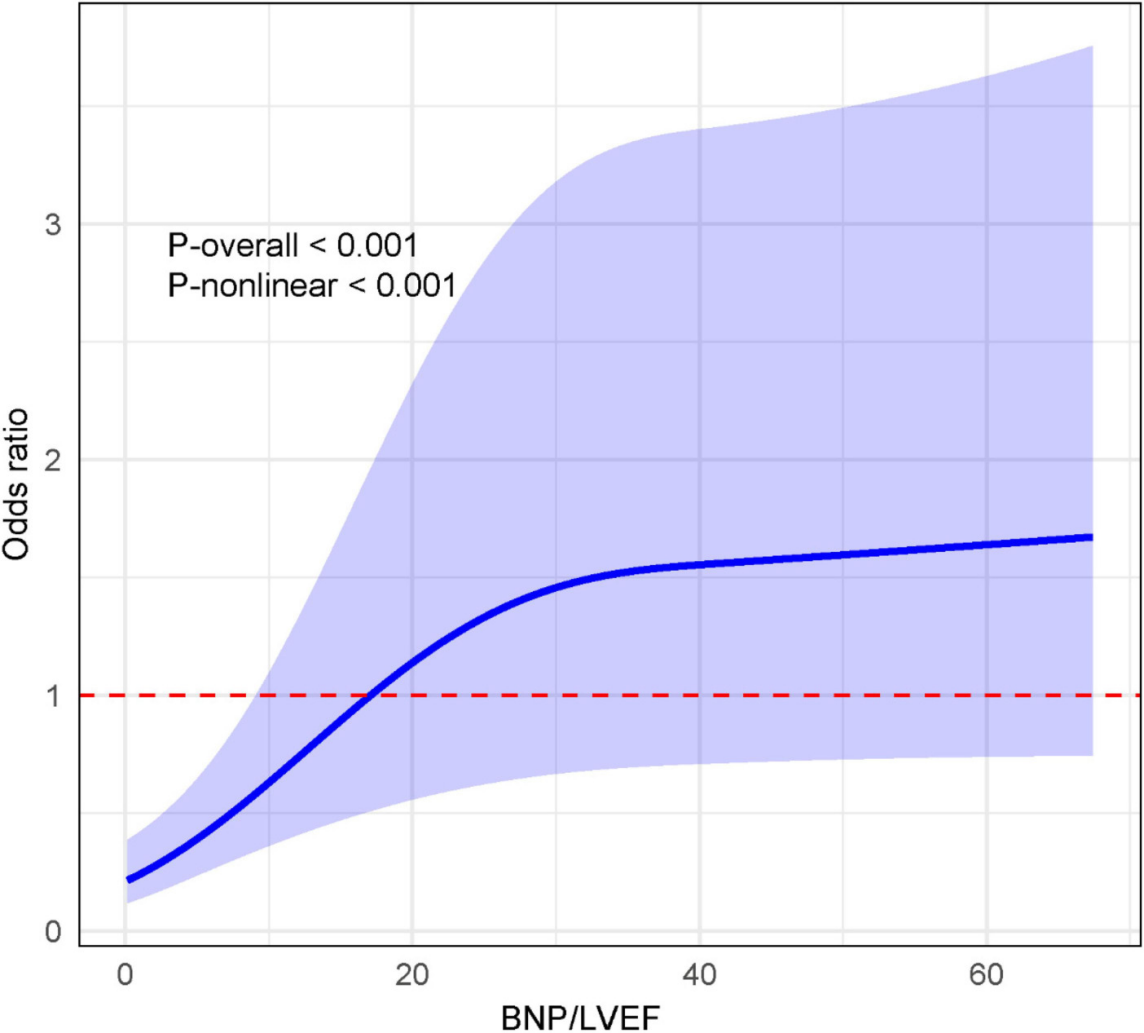
Associations between BNP/LVEF and atrial fibrillation by RCS model.

Quartile-based analyses suggested a potential nonlinear association between BNP/LVEF and atrial fibrillation. Therefore, RCS model was applied to evaluate the dos-response relationship (Figure 2). The analysis demonstrated a statistically significant nonlinear association between BNP/LVEF and AF (P-nonlinear <0.001). The odds of AF increased rapidly at lower BNP/LVEF levels and then rose more gradually at higher levels, indicating an early steep increase followed by a plateauing trend.

### 3.4 Discriminative performance of BNP/LVEF

**Figure 3:**
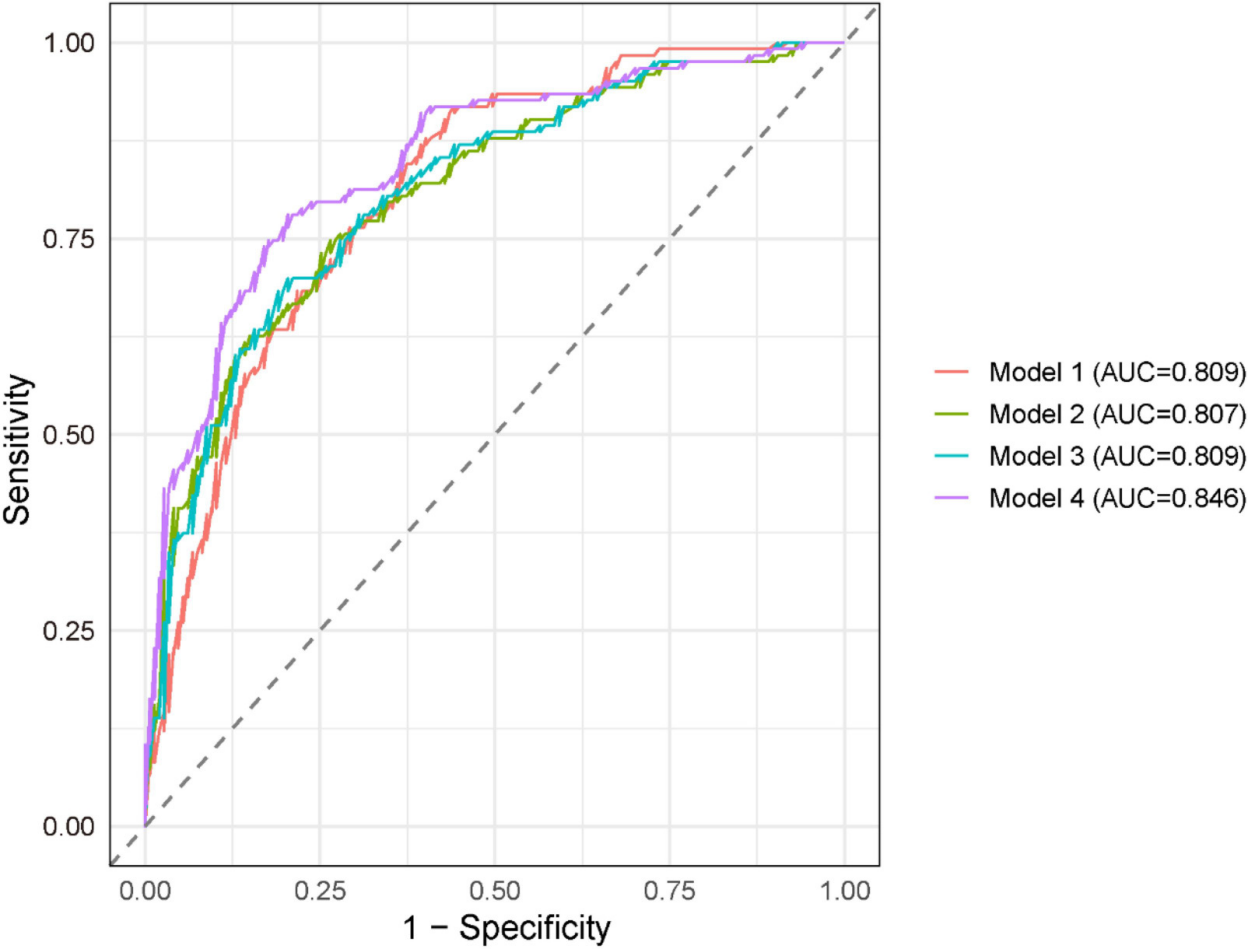
ROC curve of the relationship between BNP/LVEF and atrial fibrillation.

ROC analyses were conducted to evaluate the cross-sectional discriminative performance of BNP/LVEF for identifying prevalent atrial fibrillation during the index hospitalization across the four stepwise logistic models (Figure 3). Models 1–3 demonstrated similar discriminative ability (Model 1 AUC = 0.809; Model 2 AUC = 0.807; Model 3 AUC = 0.809). The fully adjusted Model 4 achieved a higher AUC of 0.846, indicating a modest improvement in discrimination after incorporating diabetes and CHD into the model. To further evaluate whether BNP/EF provided additional value beyond BNP or EF alone, we performed comparative ROC analyses (Supplementary Table S1; Supplementary Figure S1). BNP/EF showed an AUC of 0.846 (95% CI 0.800–0.893), which was slightly higher than the AUC for BNP alone (0.844) and EF alone (0.833). These results suggest that BNP/EF may provide modest incremental discriminative ability compared with either biomarker alone. However, the improvement over BNP alone was small, indicating broadly comparable performance among the three measures.

### 3.5 Subgroup analysis

**Figure 4:**
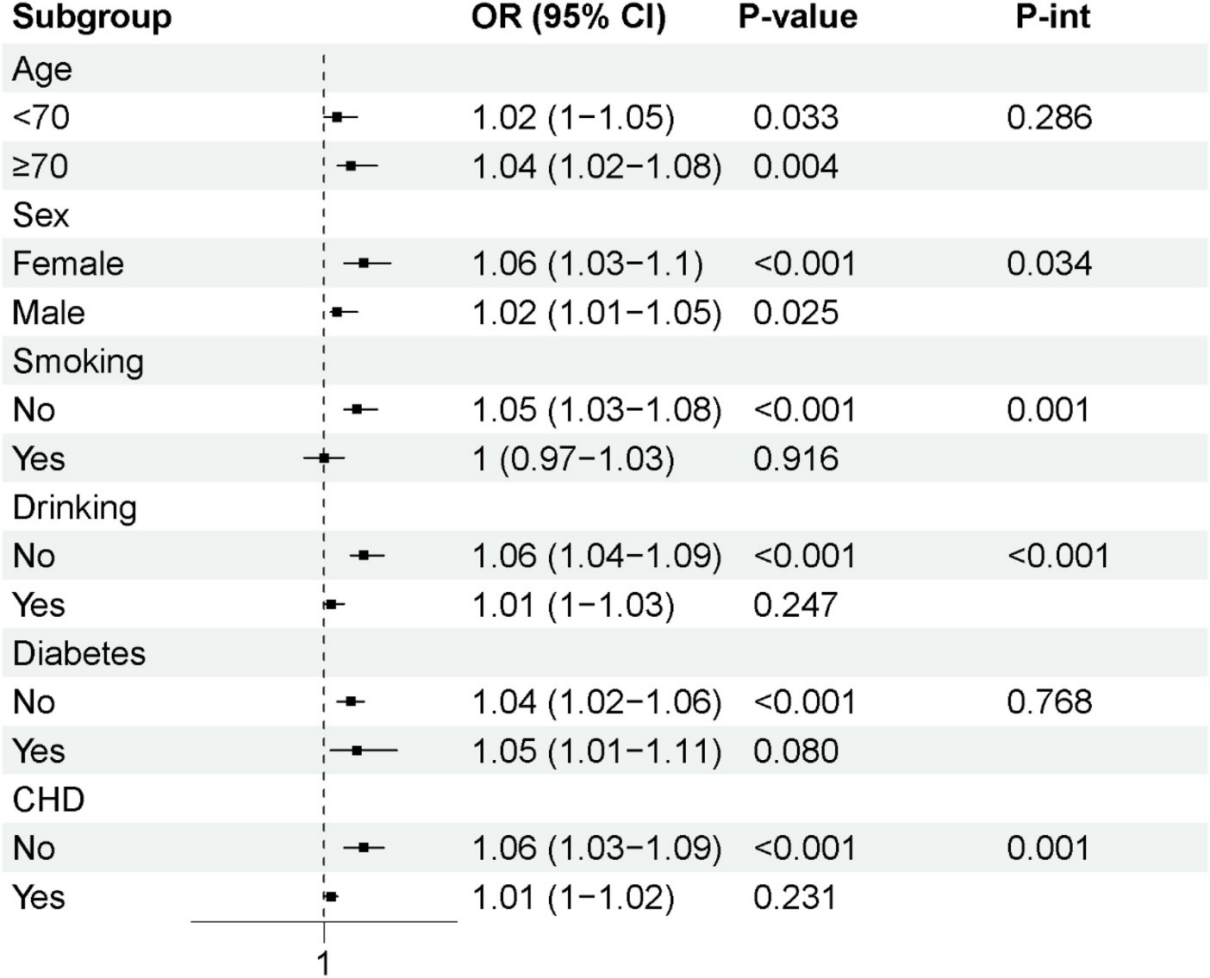
Subgroup analysis for the associations between BNP/LVEF and atrial fibrillation.

Subgroup analyses were performed according to age, sex, smoking status, alcohol consumption, diabetes, and CHD (Supplementary Table S2; Figure 4). Overall, the positive association between BNP/LVEF and AF was consistently observed across most subgroups, although significant heterogeneity was detected in several strata. The association did not differ significantly by age group or diabetes status (P-int >0.05). However, the association was stronger in females than males (female: OR 1.06, 95% CI, 1.03–1.10; male: OR, 1.02, 95% CI, 1.01–1.05; P-interaction = 0.034). The association was evident in non-smokers and non-drinkers but not in smokers or drinkers (non-smokers: OR, 1.05, 95% CI, 1.03–1.08; smokers: OR, 1.00, 95% CI, 0.97–1.03, P-interaction = 0.001; non-drinkers: OR, 1.06, 95% CI, 1.04–1.09; drinkers: OR, 1.01, 95% CI, 1.00–1.03; P-interaction <0.001). The association was present in participants without CHD (OR, 1.06, 95% CI, 1.03–1.09) but attenuated in those with CHD (OR, 1.01, 95% CI, 1.00–1.02), with a significant interaction (P-interaction <0.001).

## 4 Discussion

In this real-world, hospital-based study, we found that the integrated biomarker-phenotype index BNP/LVEF was independently and positively associated with prevalent atrial fibrillation during the index hospitalization. Across stepwise logistic regression models, BNP/LVEF remained significantly associated with AF after adjustment for demographic characteristics and cardiovascular risk factors. Dose-response analyses using restricted cubic splines further suggested a significant nonlinear relationship. BNP/LVEF also demonstrated good cross-sectional discriminative performance for identifying AF in this hospitalized sample, with slightly improved discrimination in the fully adjusted model. Subgroup analyses indicated that the association was generally consistent across age and diabetes strata but appeared more pronounced among females, non-smokers, non-drinkers, and patients without coronary heart disease, suggesting potential effect modification by sex and cardiovascular risk profiles.

In the present study, we found that higher BNP/LVEF was robustly associated with the presence of AF. These findings are consistent with previous evidence supporting the role of natriuretic peptides in AF pathophysiology (18, 19). Elevated BNP has been linked to atrial remodeling, hemodynamic stress, and AF progression in large observational cohorts (20, 21). For example, higher baseline BNP was associated with AF progression and adverse outcomes in the Outcomes Registry for Better Informed Treatment of Atrial Fibrillation II registry (11). Furthermore, studies in specific populations, such as patients with stroke or hemodialysis, have shown that increased BNP levels are associated with new AF detection or higher AF incidence, supporting the utility of BNP as a predictor for AF occurrence (22, 23). Although BNP and LVEF have each been demonstrated individually as markers of cardiac dysfunction and adverse outcomes in cardiovascular disease, including AF and heart failure (24, 25). Few studies have examined their integrated value for AF risk stratification. BNP reflects myocardial wall stress and neurohormonal activation, while LVEF quantifies left ventricular systolic performance (26). Their combination may thus capture complementary dimensions of cardiac dysfunction that predispose to AF beyond what either measure provides alone. This conceptual synergy has been shown in other cardiovascular contexts where combined biomarkers and echocardiographic indices add prognostic information beyond individual parameters (27, 28). Our findings extend these observations by demonstrating that an integrated index of BNP/LVEF is significantly associated with AF. This relationship is not fully explained by conventional risk factors. Given that elevated BNP has been implicated in atrial structural remodeling and that reduced LVEF is linked to altered hemodynamics and increased atrial pressure, the BNP/LVEF ratio may reflect underlying pathophysiological processes contributing to AF development.

In subgroup analyses, the association between BNP/LVEF and atrial fibrillation appeared broadly consistent after stratification by age and diabetic status, although these findings should be interpreted cautiously given the limited sample size. This pattern may suggest that the relationship between BNP/LVEF and atrial fibrillation is not strongly modified by age or glycemic status. This is consistent with previous evidence that natriuretic peptides reflect stress and structural remodeling of the heart itself, rather than the influence of metabolic factors alone (18). The stronger association observed in women compared to men is biologically plausible. Under similar cardiac load, women typically have higher levels of natriuretic peptides than men, which may be related to sex differences in the myocardial response to hemodynamic load and neurohormonal regulation (29). Estrogen influences natriuretic peptide expression and may interact with cardiac electrical remodeling, leading to sex differences in atrial fibrillation risk profiles (30). Furthermore, sex differences in atrial structural, electrophysiological, and fibrotic remodeling have been reported, which may exacerbate women's susceptibility to atrial fibrillation in the context of elevated BNP/LVEF (31). Notably, the association between BNP/LVEF and atrial fibrillation was significant in non-smokers and non-drinkers, but not in smokers or drinkers, and a significant interaction was observed (32). Smoking and alcohol consumption are independent risk factors for atrial fibrillation, with mechanisms including oxidative stress, autonomic dysfunction, atrial fibrosis, and direct electrophysiological disturbances (33). In smokers or drinkers, these strong external arrhythmogenic factors may mask the incremental predictive value of BNP/LVEF, thus weakening its association with atrial fibrillation in these subgroups (34). This interaction highlights the complex interplay between lifestyle factors and intrinsic cardiac remodeling in the pathogenesis of atrial fibrillation. We also observed that the association between BNP/LVEF and atrial fibrillation was maintained in participants without coronary heart disease (CHD), but this association was significantly weakened in participants with CHD. CHD leads to myocardial ischemia, fibrosis, and left ventricular systolic and diastolic dysfunction, all of which are powerful and independent drivers of elevated BNP levels and atrial fibrillation (35, 36). In the presence of CHD, the higher baseline risk of atrial fibrillation may dilute the relative contribution of BNP/LVEF as a stratification marker, leading to a significant weakening of the association (37, 38). Furthermore, CHD-related ischemic remodeling may alter natriuretic peptide secretion dynamics and ventricular mechanics, and these changes may not be fully reflected by the BNP/LVEF ratio alone. It needs to be clarified that the association between BNP/LVEF and atrial fibrillation appeared broadly consistent across age and diabetes strata in this sample. However, given the relatively small overall sample size and the limited number of participants in some subgroups, particularly smokers and drinkers, these findings should be interpreted cautiously. The observed subgroup differences may reflect sampling variability and limited statistical power rather than true biological effect modification. Accordingly, these subgroup analyses are best regarded as exploratory and hypothesis-generating.

Our research findings can be explained by several biological mechanisms. BNP is a response to increased myocardial wall stress and intracavitary pressure, leading to increased synthesis (36). Elevated circulating levels indicate ventricular and atrial pressure overload and enhanced neurohormonal compensatory activity. Simultaneously, reduced LVEF signifies impaired contractile pump function and altered ventricular biomechanics. Elevated BNP not only reflects pressure/volume overload but also actively participates in cardiovascular regulation through vasodilation, natriuresis, and inhibition of the renin-angiotensin-aldosterone system (RAAS) and the sympathetic nervous system (SNS) (39). Sustained elevated BNP is often accompanied by myocardial structural changes, such as fibrosis, chamber dilation, and extracellular matrix remodeling, which create conditions for abnormal conduction and re-entry, leading to atrial arrhythmias (36). Reduced LVEF is also associated with impaired left atrial (LA) emptying function and elevated left atrial pressure, further promoting atrial dilation and ectopic activity (40). Therefore, the BNP/LVEF ratio can comprehensively reflect pressure overload and impaired ventricular function, potentially capturing the pathophysiological environment conducive to atrial fibrillation.

ROC curve analysis indicated that BNP/LVEF demonstrated good cross-sectional discriminative performance for identifying prevalent atrial fibrillation during the index hospitalization, with relatively high AUC values in both unadjusted and partially adjusted models. The AUC values showed minimal change from Model 1 to Model 3, suggesting that the discriminatory power of BNP/LVEF primarily stems from its intrinsic characteristics and is not significantly affected by adjustments for demographic or lifestyle factors. The further increase in AUC values observed in the fully adjusted model indicates that incorporating clinical comorbidities, particularly diabetes and coronary heart disease, provides complementary prognostic information and enhances overall risk discrimination. In summary, these findings support BNP/LVEF as a reliable and clinically meaningful biomarker for atrial fibrillation risk stratification, and its cross-sectional discriminative performance can be further optimized when combined with established cardiovascular metabolic risk factors.

This study is innovative in integrating BNP and LVEF into a single composite index to evaluate atrial fibrillation risk, thereby capturing both neurohormonal activation and ventricular systolic function within a unified framework. By demonstrating a robust and nonlinear association between BNP/LVEF and AF, this work extends beyond conventional linear analyses and identifies an early risk-sensitive range that may be clinically relevant. Moreover, the consistent discriminative performance and the presence of meaningful subgroup heterogeneity highlight the potential of BNP/LVEF as a pragmatic and individualized biomarker for pragmatic marker for identifying patients with AF in hospitalized settings. Nevertheless, these findings should be interpreted as exploratory and hypothesis-generating, and further validation in prospective studies is needed to determine their potential clinical utility.

Although our findings suggest that BNP/LVEF is associated with atrial fibrillation in hospitalized patients, several issues should be considered when interpreting the generalizability of the results. First, the cross-sectional study design prevents causal inference and the determination of the temporal relationship between elevated BNP/left ventricular ejection fraction and the occurrence of atrial fibrillation. Second, this was a single-center study conducted in one tertiary hospital, and the study population may therefore reflect local referral patterns, clinical practice, and patient characteristics that are not fully representative of other hospitals or broader community settings. Second, the relatively modest sample size may have reduced the precision of effect estimates and limited statistical power, particularly in subgroup analyses, making some observed interactions exploratory rather than definitive. Because the study was restricted to a real-world hospitalized cohort, the findings may not be directly generalizable to outpatient populations, asymptomatic individuals, or patients with different disease spectra. Therefore, although the present study provides preliminary evidence supporting the association between BNP/LVEF and atrial fibrillation, external validation in larger, multicenter cohorts is needed before these findings can be widely generalized or translated into routine clinical practice. Third, BNP and left ventricular ejection fraction were measured at only a single time point, thus preventing the assessment of their dynamic changes over time. Fourth, although we adjusted for several important demographic and cardiovascular risk factors, the set of covariates available in this retrospective hospital-based dataset was limited. Therefore, residual confounding cannot be excluded, and the observed associations should be interpreted cautiously. Nonetheless, the consistency of the association across stepwise adjustment models suggests that the main findings were relatively robust within the constraints of the available data. Finally, atrial fibrillation status was assessed during hospitalization, and asymptomatic or paroxysmal atrial fibrillation may have been missed, leading to misclassification.

## 5 Conclusions

This study demonstrates that a higher BNP/LVEF ratio is independently associated with an increased likelihood of prevalent atrial fibrillation during hospitalization. This association remained robust after multivariable adjustment and exhibited a significant nonlinear dose–response relationship, with notable heterogeneity across key clinical subgroups. The BNP/LVEF ratio also showed good cross-sectional discriminative performance for identifying AF, with modest improvement when combined with cardiovascular metabolic comorbidities. In summary, these findings suggest that the BNP/LVEF ratio may serve as a practical composite marker reflecting cardiac stress and ventricular dysfunction, with potential value for identifying patients with concurrent AF in hospitalized settings. However, given the cross-sectional design, these results should be interpreted as associative rather than predictive, and further validation in prospective studies is warranted.

## Data Availability

The original contributions presented in the study are included in the article/Supplementary Material, further inquiries can be directed to the corresponding author.
