# Supplementary material for: Integrated biomarkers and cardiac phenotypes associated with atrial fibrillation: evidence from real-world hospital data
Source: Front Cardiovasc Med. 2026 May 15;13:1821154. doi: 10.3389/fcvm.2026.1821154 (PMC13218885; doi:10.3389/fcvm.2026.1821154)
Supplement: Supplementary file 1 [file Datasheet1.docx]

**Integrated biomarkers and cardiac phenotypes associated with atrial fibrillation：evidence from real-world hospital data**

Xuli Chen ^1^, Yanxi Ning ^2^, Yuxiang Wang ^3^, Yuelin Hu ^1^, Yanchao Liu ^1 *^, Wenwen Xiao ^4 *^

1. Department of Electrocardiology, The Second Affiliated Hospital of Wannan Medical University, Wuhu, China.

2. School of Pharmacy, Wannan Medical University, Wuhu, China

3. Department of Medical Service, The Second Affiliated Hospital of Wannan Medical University, Wuhu, China.

4. Eastern Theater Command Centers for Disease Control and Prevention, Nanjing, China.

**^*^Corresponding Author:**

Wenwen Xiao, Eastern Theater Command Centers for Disease Control and Prevention, 293 Zhongshan East Rd, Nanjing, China and Qiuyu Wang, Department of Electrocardiology, The Second Affiliated Hospital of Wannan Medical College, Wuhu 241000, Anhui, China.

**E-mail addresses:**

[wenwenxiao1996@163.com](mailto:wenwenxiao1996@163.com); lyc151785260031126@163.com

Table S1. Cross-sectional discriminative performance of EF, BNP, and BNP/EF for identifying prevalent atrial fibrillation during hospitalization.

| Model | AUC | lower | upper | AU (95% CI) |
| --- | --- | --- | --- | --- |
| EF | 0.833 | 0.784 | 0.882 | 0.833 (0.784-0.882) |
| BNP | 0.844 | 0.796 | 0.891 | 0.844 (0.796-0.891) |
| BNP/EF | 0.846 | 0.800 | 0.893 | 0.846 (0.800-0.893) |

Table S2. Subgroup analysis for the associations between BNP/LVEF and atrial fibrillation.

| Subgroup | N | OR | OR (95% CI) | P value | P-int |
| --- | --- | --- | --- | --- | --- |
| Age |  |  |  |  |  |
| <70 | 98 | 1.02 | 1.02 (1-1.05) | 0.033 | 0.286 |
| >=70 | 172 | 1.04 | 1.04 (1.02-1.08) | 0.004 |  |
| Sex |  |  |  |  |  |
| Female | 125 | 1.06 | 1.06 (1.03-1.1) | <0.001 | 0.034 |
| Male | 145 | 1.02 | 1.02 (1.01-1.05) | 0.025 |  |
| Smoking |  |  |  |  |  |
| No | 245 | 1.05 | 1.05 (1.03-1.08) | <0.001 | 0.001 |
| Yes | 25 | 1 | 1 (0.97-1.03) | 0.916 |  |
| Drinking |  |  |  |  |  |
| No | 246 | 1.06 | 1.06 (1.04-1.09) | <0.001 | <0.001 |
| Yes | 24 | 1.01 | 1.01 (1-1.03) | 0.247 |  |
| Diabetes |  |  |  |  |  |
| No | 228 | 1.04 | 1.04 (1.02-1.06) | <0.001 | 0.768 |
| Yes | 42 | 1.05 | 1.05 (1.01-1.11) | 0.080 |  |
| CHD |  |  |  |  |  |
| No | 171 | 1.06 | 1.06 (1.03-1.09) | <0.001 | 0.001 |
| Yes | 99 | 1.01 | 1.01 (1-1.02) | 0.231 |  |


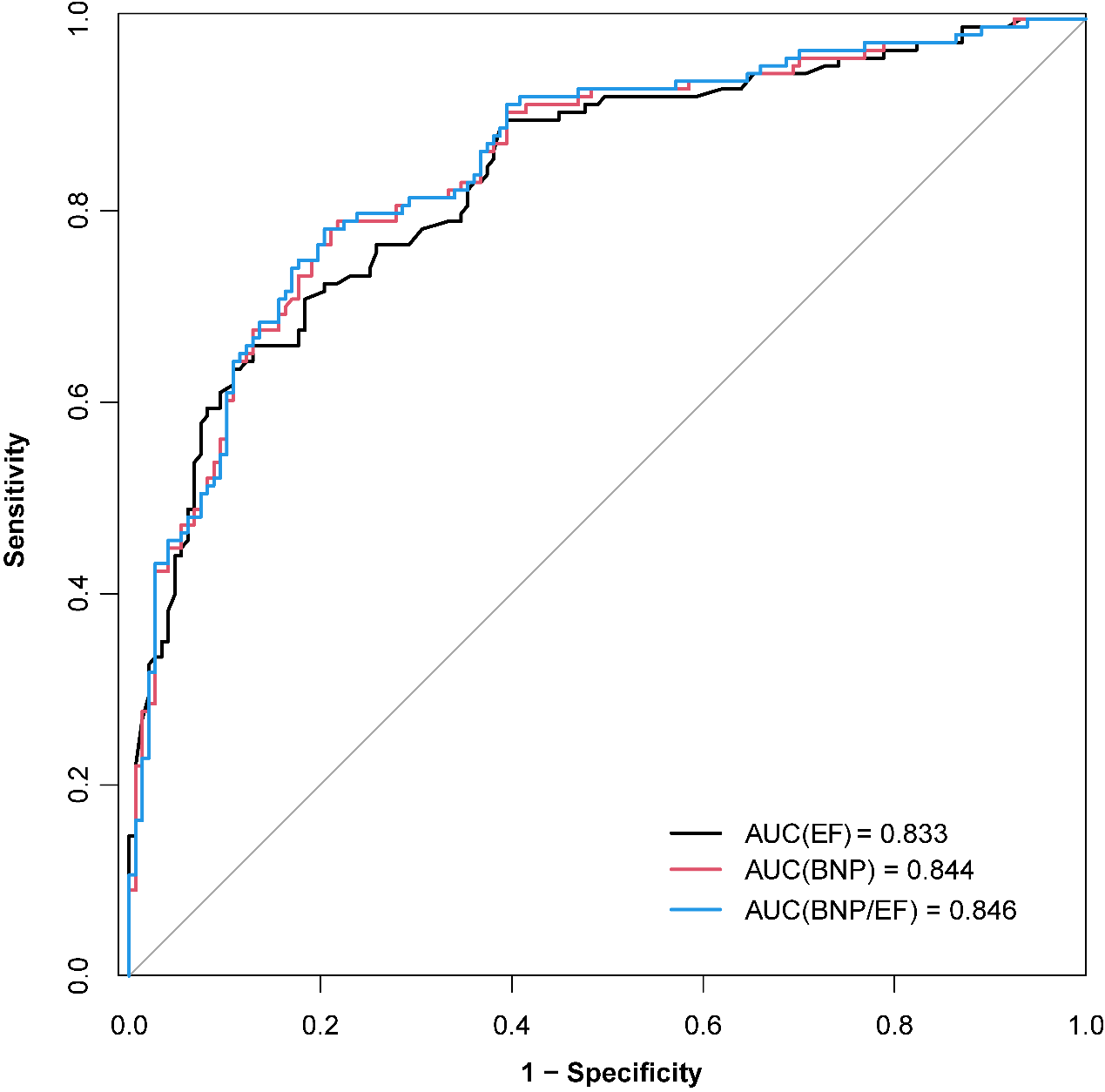


Fig. S1. Receiver operating characteristic (ROC) curves of EF, BNP, and BNP/EF for identifying prevalent atrial fibrillation in hospitalized patients.
